# Electronic versus paper diaries: a pilot study of concordance and adherence in head and neck cancer patients receiving radiation therapy

**DOI:** 10.1186/1758-3284-2-29

**Published:** 2010-10-18

**Authors:** Joseph M Blondin, Khamis S Abu-Hasaballah, Howard Tennen, Rajesh V Lalla

**Affiliations:** 1 School of Dental Medicine, University of Connecticut Health Center, Farmington, CT, USA; 2 Department of Information Technology, University of Connecticut Health Center, Farmington, CT, USA; 3 Department of Community Medicine and Healthcare, University of Connecticut Health Center, Farmington, CT, USA; 4 Department of Oral Health and Diagnostic Sciences, University of Connecticut Health Center, Farmington, CT, USA

## Abstract

**Background:**

Interactive Voice Response Systems (IVRS) and other electronic data collection methods have begun to replace conventional paper diaries as a way to capture daily patient reports. However, these methods have not been compared in head and neck (H&N) cancer patients receiving radiation therapy.

**Methods:**

15 subjects with H&N cancer were asked to complete daily IVRS calls and daily paper diaries during radiation therapy. We compared response consistency and comparative adherence across the two methods.

**Results:**

86.1% (1920/2231) of participants' responses were consistent between IVRS and paper diaries. 79.5% of the expected number of paper diaries were completed, compared to 66.2% of IVRS phone calls.

**Conclusions:**

In this pilot study of H&N cancer patients undergoing radiation therapy, concordance was high between responses recorded by paper diaries and IVRS. Although adherence appeared to be higher for the paper diaries, it is possible that they may not have been completed at the proper time.

## Background

Electronic data collection methods such as Interactive Voice Response Systems (IVRS) are becoming increasingly popular in clinical research [1]. IVRS allow clinical research participants to answer automated survey questions over the telephone, either by pressing the telephone keypad or by issuing a voice response, where the responses are validated and stored in a database for later retrieval and analysis.

Advantages of IVRS include: consistency of survey administration, real time or close to real time data collection and storage, automated reminder calls, and the ability to restrict the time window during which the IVRS survey is available to study participants [1]. However, IVRS requires training and surveys may initially take longer to complete than corresponding paper diaries. Furthermore, there may be an additional cost associated with creating and maintaining an IVRS.

Due to the increasing popularity of IVRS, it has become important to know how IVRS and conventional paper diaries compare with respect to concordance of the collected data and adherence to each data collection method. Lauristen and colleagues compared completion rates of paper, IVRS, and telephone, diaries in which subjects were asked to record symptoms of gastroesophageal reflux disease twice daily. They found that the completion rate for paper diaries was significantly higher than IVRS or the telephone diary [2]. However, there was a higher autocorrelation of consecutive entries in paper diaries, indicating possible backfilling, and as a result they concluded that although paper diaries seem to yield a higher adherence rate, electronic diaries may improve the quality of data gathered. On the other hand, Weiler et al. compared data collected using both paper and IVRS from 87 adults with allergic rhinitis and found that the data obtained by these two methods were indistinguishable from one another [3]. Lundy and Coons demonstrated the measurement equivalence of IVRS and paper forms of a widely used instrument -the EuroQol-5D- for measuring health outcomes [4]. Other studies have established equivalence for paper and electronic versions of instruments such as the Changes of Sexual Functioning Questionnaire (CSFQ) [5]; and self-report diaries in a population of drug-addicted and alcohol-dependent individuals [6]. A study of orthopedic clinic patients who completed the Short Musculoskelatal Function Assessment (SMFA) using both paper and IVRS found no significant differences in individual subject responses between the two methods [7]. However, the IVRS completion rate was significantly less than the paper completion rate.

After reviewing the available literature, Tennen et al proposed that paper diaries can be effective in situations where behaviors being studied are discrete, when same-day relationships are informative, if a lagged effect across days is expected, or if the end of day recall experience associated with paper diaries is desirable. On the other hand, electronic diaries are preferable when examining within-day temporal dynamics and when evaluating rapidly changing temporal phenomena. They also pointed out that the characteristics of the study population can influence the acceptability of electronic diaries, such as in older populations [8].

In this context, patients undergoing radiation therapy for head and neck cancer represent a unique population with significant morbidity associated not only with the underlying disease but also with the treatment (radiation therapy). There has been a large increase in clinical research in this population, addressed to treatment of the cancer and the side-effects of radiation therapy, notably mucositis [9-11]. In addition, routine clinical care of these patients also involves collection of data to monitor pain, side-effects, etc. It therefore becomes important to know how paper and electronic diaries, including IVRS, compare in this population so that future research studies and clinical data collection can be appropriately designed.

## Methods

Fifteen head and neck cancer patients, participating in an IRB-approved clinical trial of an anti-inflammatory medication for radiation-induced oral mucositis, completed IVRS and paper diaries daily over a six to seven week treatment period [12]. Written informed consent was obtained from all patients. Subjects were trained on how to complete the paper and IVRS diaries. They were instructed to complete the paper diary anytime during the day before bedtime and the IVRS telephone call before 7 pm. They were not instructed in which order to complete the diaries. Subjects who failed to complete the IVRS call by 7 pm received an automated telephone call reminding them to complete their daily IVRS diary and offered the option to connect them with the IVRS in order to complete the diary at that time. They received verbal reminders to complete the paper diaries when they came in for study visits. There was no financial compensation for completing the diaries. Onsite study visits took place every 2-3 days over this period, during which the subject was examined and the paper diaries were collected.

The paper and IVR diaries asked the same 10 questions (listed in Table 1). The three primary questions, directed to compliance with study medication, side-effects, and concomitant medications, were asked of all subjects. The secondary questions were asked only if a subject answered "yes" to a related primary question, to get additional detail on study medication use, side-effects, and/or new concomitant medications.

**Table 1:** Concordance rates, between paper diaries and Interactive Voice Response diaries, for individual questions across all subjects

	Consistent (%)	Inconsistent (%)
**Primary Questions**		

Did you take your study medication today?	98.9	1.1

Did you experience any side effects today?	84.8	15.2

Did you take any additional medication today?	73.4	26.6

**Secondary Questions**		

What time did you take your study medication?	87.7	12.3

What time did you experience a side effect?	100	0

What was the side effect?	100	0

What time did you take the additional medication?	81.3	18.7

What is the name of the additional medication?	100	0

What dosage was the additional medication?	68.3	31.7

Why did you take the additional medication?	84.5	15.5

Both the IVRS and paper diaries allowed participants to report more than one side effect and more than one additional medication. Some of the IVRS secondary questions required a spoken response, whereas others required keypad entry. Spoken responses were recorded and later transcribed for analysis.

To evaluate the concordance of responses collected via paper and IVRS diaries, the number of consistent and inconsistent responses for every diary question was calculated using the following pre-defined criteria:.

• For the 3 primary questions, which required a "yes/no" response, an identical response across the two methods was considered consistent.

• For the question on the time of taking study medication, times within 30 minutes of each other were considered consistent.

• For the questions on the description of side-effects and reason for taking additional medications, consistency grade was assigned by reviewers based on comparison of the verbal responses.

• For the question requesting the name of the concomitant medication, the data was considered consistent if responses via both methods of data entry contained the brand or generic name of the same drug.

• For the question regarding dosage of concomitant medications, exactly the same dosage was considered consistent.

Using these criteria, concordance rates for each question across all subjects, and concordance rates for each subject across all questions were calculated. In addition, adherence with diary completion, for paper as well as IVRS, was calculated using the number of completed diaries as a percentage of the total number expected for each subject.

## Results

### Concordance

**Figure 1:**
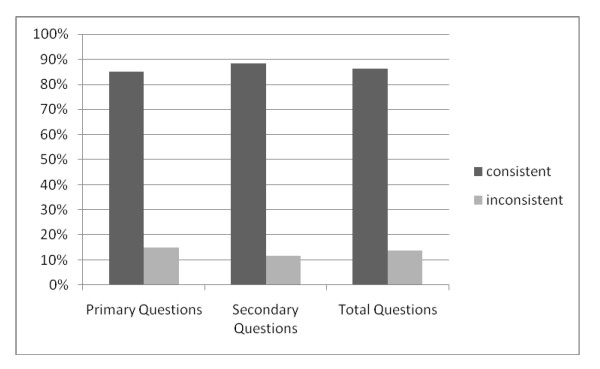
**Concordance between Paper Diaries and Interactive Voice Response Diaries**.

Two reviewers independently rated a randomly generated sample of 20% of participants' diary responses on days on which both a paper diary and an IVRS call was completed. The two reviewers rated 358 of 360 responses consistently across the electronic and paper diary methods (Kappa = 0.99). Concordance was calculated based on a comparison of IVRS and paper diary responses for each day that both were completed. The analysis revealed that 86.1% (1920/2231) of patient responses were consistent across IVRS and paper diaries. Comparison of the primary and secondary questions showed that 85% (1261/1484) of responses to primary questions and 88.2% (659/747) of responses to secondary questions were consistent (see Table 1 and Figure 1). Eight of the 15 study participants showed concordance rates > 90% (see Table 2). Two subjects had concordance rates under 80%. One of these subjects recorded many side-effects on the paper diary, but reported very few side-effects via the IVRS, leading to low concordance. The other subject reported a large number of days with concomitant medications on the paper diary but considerably fewer on the electronic diary.

**Table 2:** Concordance rates, between paper diaries and Interactive Voice Response (IVR) diaries, for individual subjects

Subject Number	Percentage of questions to which responses were consistent between paper diaries and IVR.	Percentage of questions to which responses were inconsistent between paper diaries and IVR.
1	80.2	19.8

2	81.8	18.2

3	72.6	27.4

4	94.6	5.4

5	94.1	5.9

6	77.6	22.4

7	80.8	19.2

8	93.3	6.7

9	82.1	17.9

10	97.9	2.1

11	91.2	8.8

12	85.1	14.9

13	98.6	1.4

14	92.6	7.4

15	94.9	5.1

### Adherence

**Figure 2:**
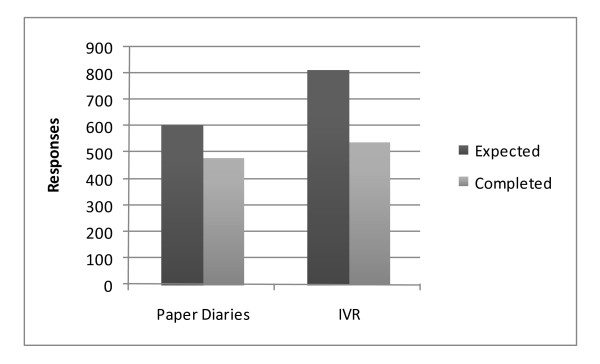
**Adherence data for Paper Diaries and Interactive Voice Response (IVR) Diaries**.

Instrument-based adherence for the entire sample (i.e. total number of each diary completed for all subjects as compared to number expected) was higher for the paper diaries (481 completed paper diaries of 605 expected, 79.5%) compared to the IVRS diaries (540 completed phone calls of 816 expected, 66.2%) (p < 0.001) (see Figure 2). The expected total number of completed IVRS calls was higher than the expected total number of completed paper diaries because the first four subjects were asked to make their IVR calls twice daily while all subjects were asked to do the paper diary only once daily. After accounting for the correlations in completion rates within subjects, analysis of subject-based adherence revealed that the odds that an individual subject would complete a paper diary were 1.91 times the odds that the same subject would complete an IVRS phone call (p < 0.001; 95% confidence interval (1.68, 2.19)).

There were 98 days on which the paper diary was completed and IVRS was not. On these 98 days, the total number of concomitant pain medication doses used was 40 (including the same medications taken on multiple days and multiple medications taken on the same day). There were 115 days on which IVRS was completed but not the paper diary. On these 115 days, the total number of concomitant pain medication doses used was only 2.

## Discussion

We found a high level of day-to-day concordance between daily patient reports entered via IVRS and those entered via a paper record. This high level of concordance was obtained despite the fact that the specific times at which each diary was completed may have varied daily and some paper diaries may have been completed one or two days after the target day (as paper diaries were collected every 2-3 days). We caution that this level of concordance may not generalize to diary items measuring momentary emotional states or other features of daily experience that cannot be recalled reliably.

Only two diary items, "*Did you take any additional medication today*?" and "*What dosage was the additional medication*?" yielded less than 80% concordance. These are also the responses most likely to vary from day to day. It is also possible that some subjects took an additional medication after completing the IVRS call for the day, but reported the additional medication on that day's paper diary (since the paper diary was accessible to the subject till handed in at the next study visit). Furthermore, subjects who had trouble speaking due to radiation-induced oral ulcerations may have preferred to select a negative response that required pressing the phone keypad but not a subsequent voice response.

Previous studies comparing adherence to IVRS to that for paper diaries have yielded mixed results [2,13]. In this study, we found greater adherence with the paper diaries. Several factors may explain this finding. The predominantly older head and neck cancer population may not be as adept at or willing to use an automated IVR system [14]. In addition, most patients receiving radiation therapy for head and neck cancer develop severe mouth and/or throat ulcerations (mucositis) that can make it very painful to speak. This may have led to some reluctance to complete the IVRS calls, which in some cases required verbal responses. This explanation is supported by our finding of greater pain medication use (indicating more severe mucositis) on days on which the paper diary was completed but not the electronic diary. Another potential concern is that subjects having difficulty speaking may have preferred to answer "No" to the primary questions so as to avoid answering secondary questions, some of which required spoken responses. Thus, there may have been an unintentional "response cost" associated with a "Yes" response to the primary questions. While it is difficult to control for such behavior, if it did occur, it would argue against use of IVRS with verbal responses in this population. It is note-worthy that our use of IVRS to record verbal responses is unusual and is not a feature of many commercial IVR systems.

A limitation of this study, in addition to our small sample, was that it compared a sophisticated IVR system, including automated reminders and time stamped data entry verification, to a paper diary for which on-time data entry was not verified. Thus, there is a possibility that some paper diaries may have been backfilled a day or two later [4]. However, our high level of day-to-day cross-method concordance, and collection of paper diaries at study visits every 2-3 days, argues against significant backfilling. It is also possible that subjects may have referred to the paper diaries while responding to the IVRS questions. However, this is unlikely since subjects were provided a separate handout with IVRS instructions and access codes, which they needed to refer to while making the IVRS calls.

## Conclusion

In conclusion, in this pilot study of head and neck cancer patients receiving radiation therapy, we observed high concordance between paper diaries and IVRS, and lower adherence to IVRS. These findings have implications for the optimal design of clinical and research data collection methods in this complex population. Because other electronic data capture methods, such as personal digital assistants (PDAs) and IVR without verbal responses, would not have required spoken responses, direct comparisons of paper diaries to these electronic diary methods in comparably challenged patient samples could be revealing.

## Competing interests

The authors declare that they have no competing interests.

## Authors' contributions

All authors participated in the research design. JMB and RVL implemented the study. All authors read and approved the final manuscript.
